# Volume change with gantenerumab: Impact of amyloid‐related imaging abnormalities and amyloid removal

**DOI:** 10.1002/alz.71749

**Published:** 2026-08-14

**Authors:** Matteo Tonietto, Erica Silvestri, Christopher R. S. Belder, Frederik Barkhof, Nick C. Fox, Janice Smith, Gregory Klein

**Affiliations:** ^1^ Neuroscience and Rare Diseases Roche Pharma Early Research and Development, F. Hoffmann‐La Roche Ltd Basel Switzerland; ^2^ A4P Consulting Ltd. Sandwich UK; ^3^ Dementia Research Centre UCL Queen Square Institute of Neurology London UK; ^4^ Department of Neurology Royal Adelaide Hospital and Queen Elizabeth Hospital Adelaide South Australia Australia; ^5^ School of Medicine Adelaide University Adelaide South Australia Australia; ^6^ Department of Radiology & Nuclear Medicine Amsterdam UMC, Vrije Universiteit Amsterdam the Netherlands; ^7^ Roche Products Ltd Welwyn Garden City UK

**Keywords:** Alzheimer's disease, amyloid, anti‐amyloid, brain volume changes, gantenerumab

## Abstract

**INTRODUCTION:**

Amyloid‐lowering antibodies are associated with neuroanatomical volume changes. This study aims to disentangle the contributions of amyloid‐related imaging abnormalities (ARIA) and amyloid clearance to these changes.

**METHODS:**

Data from the GRADUATE I and II trials of gantenerumab in early Alzheimer's disease were analyzed. Participants were grouped as placebo, gantenerumab‐treated without ARIA, or gantenerumab‐treated with ARIA.

**RESULTS:**

Gantenerumab treatment was associated with greater parenchymal volume reduction than placebo, with no difference between ARIA and non‐ARIA groups. The ARIA group exhibited greater ventricular enlargement which was offset by reduced external cerebrospinal fluid space expansion. Greater amyloid removal correlated with greater volume reduction, although volume changes also occurred in white matter, where amyloid is minimal. Furthermore, the ARIA group showed a flatter relationship between ventricular expansion and clinical decline.

**DISCUSSION:**

ARIA are not associated with long‐term parenchymal reduction; rather, they are linked to a fluid shift from the external space to the ventricles. Amyloid removal correlates with parenchymal changes, but white matter changes suggest additional mechanisms beyond local clearance. Neither phenomenon was associated with worse clinical outcomes.

**TRIAL REGISTRATION NUMBER:**

NCT03444870 and NCT03443973

## Highlights

- Treatment with gantenerumab is associated with parenchymal volume reduction, and ventricular enlargement.
- Amyloid‐related imaging abnormalities (ARIA) do not drive long‐term parenchymal brain volume reduction.
- ARIA are associated with a fluid shift from the external cerebrospinal fluid spaces to the ventricles.
- Parenchymal volume reduction is spatially linked to the magnitude of local amyloid clearance.
- Neither ventricular enlargement nor parenchymal volume reduction was associated with worse clinical outcomes.

## 1 BACKGROUND

Amyloid‐targeting monoclonal antibodies are a class of therapies for Alzheimer's disease (AD) designed to clear pathogenic amyloid beta (Aβ) plaques from the brain. Late‐stage clinical trials for several agents in this class, including aducanumab,^1^ bapineuzumab,^2^ donanemab,^3^ gantenerumab,^4^ and lecanemab^5^ have demonstrated the ability to remove Aβ plaques, although the degree of amyloid removal varies between molecules. Those therapies achieving the highest levels of amyloid removal have also demonstrated statistically significant benefits across several clinical measures, leading to regulatory approval for some agents.^6^

A consistent observation across these trials is the finding of greater brain volume reduction in treated participants compared to placebo.^7^ The magnitude of these changes varied by brain region: the effect on cortical or whole brain volume was generally small and the impact on the hippocampi was variable, while the most pronounced change was ventricular enlargement.^8^ Furthermore, the magnitude of these volume changes has been shown to be positively correlated with both the extent of amyloid removal and the incidence of amyloid‐related imaging abnormalities (ARIA), a common side effect of these therapies.^7^, ^8^

Several hypotheses have been proposed to explain these volumetric changes. These include direct consequences of amyloid plaque removal and its associated cellular components,^8^, ^9^ the resolution of plaque‐related neuroinflammation,^8^, ^9^, ^10^ fluid shifts,^7^, ^8^, ^9^, ^10^, ^11^ and the possibility of treatment‐induced neurodegeneration.^7^, ^12^, ^13^ The contribution of these potential mechanisms and the validity of the underlying hypotheses remain under debate.^14^, ^15^

The phase III GRADUATE I and GRADUATE II trials assessed the efficacy and safety of gantenerumab, a fully human amyloid‐targeting monoclonal antibody, in early AD.^4^ Although the gantenerumab program was terminated due to the benefit–risk observed in this population not supporting further development, a rich dataset was collected from nearly 2000 participants followed longitudinally for up to 116 weeks. The present work made use of this extensive dataset to try to disentangle the respective impacts of ARIA and plaque removal on brain volume, aiming to clarify the key mechanisms driving these treatment‐associated changes. 

## 2 METHODS

### 2.1 Study design

GRADUATE I and II (NCT03444870, NCT03443973) are two identical global phase III, multicenter, randomized, double‐blind, placebo‐controlled, parallel‐group trials designed to evaluate the efficacy and safety of gantenerumab in participants with early AD. A total of 2033 participants were recruited from 309 sites in 30 countries. The eligibility criteria included age between 50 and 90 years and evidence of mild cognitive impairment due to AD or mild dementia due to AD. Moreover, all the participants had to be amyloid positive, measured either by amyloid positron emission tomography (PET) visual read or a cerebrospinal fluid (CSF) test using the Elecsys immunoassay. Participants were randomly assigned in a 1:1 ratio to receive subcutaneous gantenerumab or placebo. The study lasted 116 weeks, and it included longitudinal magnetic resonance imaging (MRI) assessment in every participant, as well as optional longitudinal amyloid PET assessment in a subset of participants. Additional details are provided in the protocol and statistical analysis plan in the supporting information.

### 2.2 Volumetric MRI

T1‐weighted (T1w) magnetic resonance sequences were collected in all participants at screening, week 48, week 104, and week 116. The current analysis was restricted to longitudinal images acquired on the same MRI scanner as the one used at screening. Voxel‐wise volume changes from baseline were calculated using the Jacobian integration method (Supplementary Methods in supporting information).^16^

The following regions of interest (ROIs) were defined on the baseline T1w images: cortex, further divided in cingulate, frontal, occipital, parietal, and temporal lobes; white matter; cerebellum; caudate; pallidum; putamen; thalamus, lateral, third, and fourth ventricles; choroid plexus; external CSF (i.e., part of convexity and sulcal CSF adjacent to the cortical surface); and whole CSF (lateral, third, and fourth ventricles and external CSF combined). A detailed procedure of how these regions were obtained is described in the Supplementary Methods and Table S1 in supporting information.

Regional volume changes, expressed as a percentage change from baseline, were computed by integrating the voxel‐wise Jacobian determinants within each regional mask.

### 2.3 ARIA

ARIA comprise vasogenic edema and/or sulcal effusions (ARIA‐E), which were evaluated on 2D fluid‐attenuated inversion recovery (FLAIR) sequences, and microhemorrhages and/or superficial siderosis (ARIA‐H), which were evaluated on 2D T2*‐weighted sequences by a single central‐read neurologist. Presence of ARIA was evaluated regularly during the trial, as described elsewhere.^4^, ^17^ ARIA‐E radiological severity was determined using the Barkhof Grand Total Score (BGTS).^18^ The analyses performed in this work were focused on ARIA‐E as the higher incidence of ARIA‐H in the active arm was driven by ARIA‐H concurrent with ARIA‐E, while incidence of new isolated ARIA‐H was comparable between arms.^4^

**RESEARCH IN CONTEXT**

1. **Systematic review**: Amyloid‐lowering monoclonal antibodies are consistently associated with greater neuroanatomical volume changes and ventricular enlargement compared to placebo. The biological mechanisms driving these structural differences remain unclear.
2. **Interpretation**: Using the phase III GRADUATE trials dataset, this study demonstrates that amyloid‐related imaging abnormalities (ARIA) do not drive long‐term parenchymal volume reduction. Instead, ARIA cause a fluid shift from the external cerebrospinal fluid space into the ventricles. Conversely, parenchymal volume reduction is spatially linked to the magnitude of local amyloid clearance. Neither phenomenon was associated with worse clinical or cognitive outcomes.
3. **Future directions**: Because most participants remained amyloid positive at the end of the study, this precludes conclusions about the structural effects after complete amyloid clearance. Future research must assess long‐term volumetric trajectories once complete amyloid clearance is achieved.

### 2.4 Amyloid PET

Amyloid PET scans were performed at screening, week 52, week 104, or week 116 in a subset of participants who consented to participate in the amyloid PET substudy using either [^18^F]florbetaben or [^18^F]flutemetamol. Regional standardized uptake value ratio (SUVR) values were calculated for the parenchymal ROIs listed above, using the whole cerebellum as reference region, and converted to regional Centiloid values using tracer specific Centiloid conversion equations, as detailed in the Supplementary Methods.

### 2.5 Statistical analysis

Participants were divided into three groups: placebo, gantenerumab‐treated participants who did not develop ARIA‐E, and gantenerumab‐treated participants who developed ARIA‐E.

Differences in percentage volume and regional Centiloid change from baseline between groups were assessed using mixed model repeated measures (MMRM). The model included the change from baseline (screening visit) in imaging metrics as the dependent variables, while adjusting for group (as categorical), visit (as categorical), apolipoprotein E (*APOE*) ε4 status (as categorical; carrier vs. non‐carrier), type of tracer (for amyloid PET only, as categorical), baseline Centiloid (for amyloid PET only), Centiloid‐by‐visit (for amyloid PET only), and group‐by‐visit interaction. Visit was treated as the repeated variable within a participant. Additionally, the MMRM for percentage volume change was repeated in subgroups stratified by *APOE* ε4 allele count (0, 1, or 2 alleles).

Within the gantenerumab‐treated ARIA group, the relationship between regional volume changes and ARIA‐E radiological severity (defined as the maximum BGTS recorded for each participant across all time points) was assessed with the Spearman correlation coefficient. Additionally, correlations between volume changes in different regions were assessed across all three study groups.

The relationship between volume change and amyloid removal was assessed using a region‐wise correlation analysis. For each gray matter region, the difference in the mean change from baseline between the gantenerumab and placebo groups was calculated for both volume and amyloid load from the MMRM analysis. A Spearman correlation coefficient was then computed across these regional net treatment effects. This approach was selected to specifically isolate the drug's effect, as a standard between‐subject correlation would be confounded by the larger contribution of disease progression to brain volume changes.

The association between longitudinal changes in brain volume (cortex and lateral ventricles) and clinical decline from baseline to the end of the study was assessed using linear regression models. Clinical outcomes included the study primary endpoint, Clinical Dementia Rating Sum of Boxes (CDR‐SB), and secondary endpoints: Alzheimer's Disease Assessment Scale Cognitive Subscale 13 (ADAS‐Cog 13), Alzheimer's Disease Cooperative Study Activities of Daily Living (ADCS‐ADL), and Functional Activities Questionnaire (FAQ). The models included the change in clinical score as the dependent variable, with an interaction term between percentage volume change and group included as the predictor. This formulation allowed for the estimation of group‐specific associations (slopes) and pairwise comparisons to assess whether the relationship between volume change and clinical progression differed between groups.

Due to the exploratory nature of this analysis, multiplicity adjustments were not applied to the tests.

The results presented here are based on a *de novo* image analysis and may differ numerically from previously published core lab findings from the trials.^4^, ^19^

## 3 RESULTS

### 3.1 Population

A total of 1548 participants were included in the analysis after excluding those without a screening MRI (7), a post‐baseline MRI (254), those with a change in MRI scanner (168), and those for whom the analysis pipeline failed (56; Figure S1 in supporting information). Of this cohort, 213 participants also had longitudinal amyloid PET data. Baseline characteristics, detailed in Table 1, were generally well matched across the study groups. As expected, *APOE* ε4 carriers were overrepresented in the ARIA group, consistent with *APOE* ε4 being a known risk factor for ARIA.^20^ The distribution of radiological severity of ARIA‐E in the ARIA group was as follows: 39 participants (18.8%) were classified as having a maximum severity of mild (BGTS 1–3); 122 participants (58.7%) were moderate (BGTS 4–14); and 47 participants (22.6%) were severe (BGTS 15–60; Figure S2 in supporting information).^21^

**TABLE 1:** Demographics and clinical characteristics of the analyzed population.

	All participants			Longitudinal amyloid PET subgroup		
	Placebo (*n* = 762)	Gantenerumab no‐ARIA (*n* = 578)	Gantenerumab ARIA (*n* = 208)	Placebo (*n* = 102)	Gantenerumab no‐ARIA (*n* = 77)	Gantenerumab ARIA (*n* = 34)
Age (years)						
Mean ± SD	71.5 ± 7.6	70.9 ± 8.2	70.3 ± 6.8	71.7 ± 8.2	70.7 ± 9.1	72.6 ± 6.0
Sex, *n* (%)						
F	428 (56)	321 (56)	134 (64)	49 (48)	29 (38)	25 (74)
M	334 (44)	257 (44)	74 (36)	53 (52)	48 (62)	9 (26)
*APOE* e4 allele, *n* (%)						
Carrier	515 (68)	347 (60)	175 (84)	73 (72)	51 (66)	28 (82)
Non‐carrier	247 (32)	231 (40)	33 (16)	29 (28)	26 (34)	6 (18)
Clinical stage, *n* (%)						
Mild	343 (45)	262 (45)	89 (43)	45 (44)	36 (47)	7 (21)
Prodromal	419 (55)	316 (55)	119 (57)	57 (56)	41 (53)	27 (79)
Use of medication for Alzheimer's disease symptoms, *n* (%)						
No	290 (38)	201 (35)	73 (35)	35 (34)	24 (31)	15 (44)
Yes	472 (62)	377 (65)	135 (65)	67 (66)	53 (69)	19 (56)
CDR‐SB						
Mean ± SD	3.5 ± 1.5	3.6 ± 1.6	3.6 ± 1.6	3.5 ± 1.5	3.8 ± 1.6	3.3 ± 1.2
MMSE						
Mean ± SD	23.8 ± 3.1	23.7 ± 3.1	23.5 ± 3.3	23.8 ± 3.2	23.7 ± 3.1	23.1 ± 3.5

Abbreviations: *APOE*, apolipoprotein E; ARIA, amyloid related imaging abnormalities; CDR‐SB, Clinical Dementia Rating Sum of Boxes scores; MMSE, Mini‐Mental State Examination; PET, positron emission tomography; SD, standard deviation.

### 3.2 Regional differences in volume change from baseline

Over the 116‐week study period, all three study groups exhibited continuous brain volume change from baseline.

**FIGURE 1:**
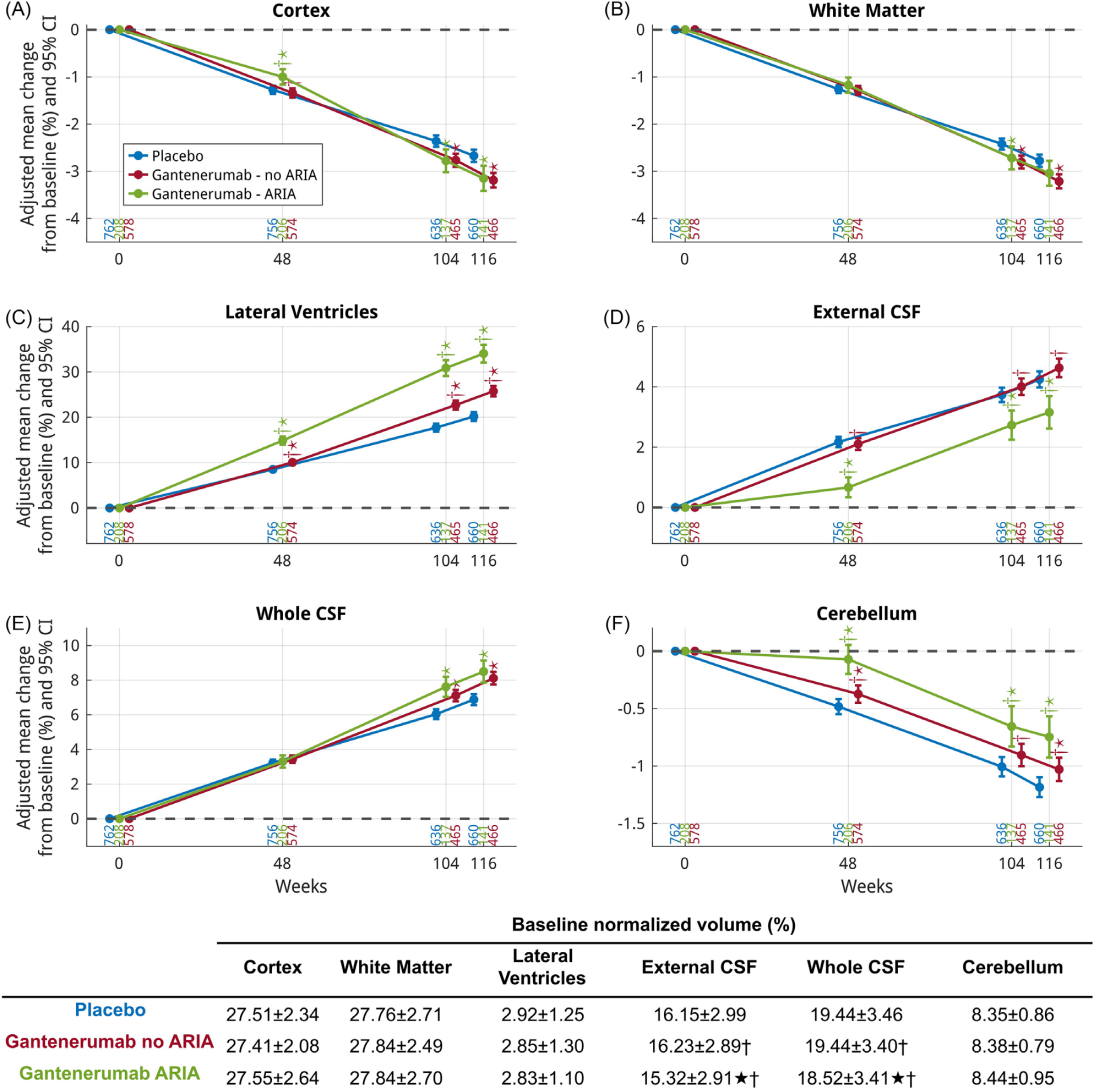
Effect of gantenerumab on longitudinal brain volume changes. This figure shows the adjusted mean percentage change from baseline with 95% CIs for various brain region volumes over 116 weeks, obtained from the MMRM analysis. The lines represent the placebo group (blue), the gantenerumab group without ARIA (red), and the gantenerumab group with ARIA (green). A star (★) denotes a statistically significant difference (*p* < 0.05) for a gantenerumab group compared to the placebo group, while a dagger (†) indicates a significant difference (*p* < 0.05) between the gantenerumab with ARIA and without ARIA groups. The embedded table provides the baseline mean and standard deviation normalized volume, which is expressed as a percentage of the estimated total intracranial volume. ARIA, amyloid‐related imaging abnormalities; CI, confidence interval; CSF, cerebrospinal fluid; MMRM, mixed model for repeated measures.

In the cortex (Figure 1A), gantenerumab treatment led to greater volume reduction compared to placebo (adjusted mean difference placebo vs. non‐ARIA: −0.52%; 95% confidence interval [CI], −0.72% to –0.32%; *p* < 0.001; vs. ARIA: −0.48%; 95% CI, −0.78% to –0.18%; *p* = 0.002). The gantenerumab ARIA group showed an early transient effect, with initially less volume reduction at week 48 compared to the other groups. At this time point, 94 of 206 participants in the ARIA group had active ARIA‐E (Table S2 in supporting information). By the end of the study, when most ARIA‐E events had resolved, cortical volume changes were no longer significantly different between the ARIA and non‐ARIA groups (adjusted mean difference: 0.04%; 95% CI, −0.27% to 0.35%; *p* = 0.81). Analysis of individual cortical lobes confirmed these findings, showing the transient effect in the ARIA group was most evident in the occipital lobe, a common site for ARIA‐E (Figure S3A‐SE in supporting information). Subcortical regions showed consistent results, with the gantenerumab ARIA group not showing greater volume reduction than the non‐ARIA group in any region (Figure S3F‐SI).

For white matter (Figure 1B), a similar pattern was observed. Compared to placebo, a significant volume reduction was seen in the non‐ARIA gantenerumab‐treated group (adjusted mean difference: −0.44%; 95% CI, −0.64% to –0.24%; *p* < 0.001), but the reduction in the ARIA group did not reach statistical significance (adjusted mean difference: −0.27%; 95% CI, −0.56% to 0.03%; *p* = 0.08). The volume change did not differ significantly between the ARIA and non‐ARIA groups (adjusted mean difference: 0.17%; 95% CI, −0.13% to 0.48%; *p* = 0.27).

In contrast, ventricular and external CSF volumes showed a different pattern (Figure 1C‐D). The gantenerumab ARIA group exhibited substantially greater lateral ventricular enlargement than the non‐ARIA group and placebo (adjusted mean difference at week 116 vs. non‐ARIA: 8.29%; 95% CI, 6.01% to 10.57%; *p* < 0.001; vs. placebo: 13.87%; 95% CI, 11.66% to 16.07%; *p* < 0.001). Correspondingly, the ARIA group had lower external CSF expansion than both other groups at all post‐baseline time points (adjusted mean difference at week 116 vs. non‐ARIA: −1.47%; 95% CI, −2.10% to –0.85%; *p* < 0.001; vs. placebo: −1.09%; 95% CI, −1.70% to –0.49%; *p* < 0.001). While the non‐ARIA group also showed greater ventricular enlargement than placebo (adjusted mean difference at week 116: 5.58%; 95% CI, 4.07% to 7.08%; *p* < 0.001), their external CSF expansion did not differ significantly (adjusted mean difference at week 116: 0.38%; 95% CI, −0.03% to 0.79%; *p* = 0.07).

For the whole CSF (Figure 1E), both gantenerumab‐treated groups exhibited greater volume expansion compared to placebo. However, the volume change did not differ significantly between the ARIA and non‐ARIA groups (adjusted mean difference: 0.39%; 95% CI, −0.36% to 1.12%; *p* = 0.31).

Finally, for the cerebellum, both gantenerumab‐treated groups experienced less volume reduction compared to placebo, with the ARIA cohort showing even less volume reduction than the non‐ARIA cohort (adjusted mean difference ARIA vs. non‐ARIA: 0.28%; 95% CI, 0.08% to 0.49%; *p* = 0.008; Figure 1F).

Subgroup analyses stratified by *APOE* ε4 allele count (0, 1, or 2 alleles) yielded results largely consistent with the overall cohort (Figures S4–S6 in supporting information).

All analyses were repeated using absolute volume changes. The overall longitudinal trends, group differences, and levels of statistical significance remained consistent across all evaluated regions (Figure S7 in supporting information).

### 3.3 Association between volume changes and ARIA severity in the ARIA group

**FIGURE 2:**
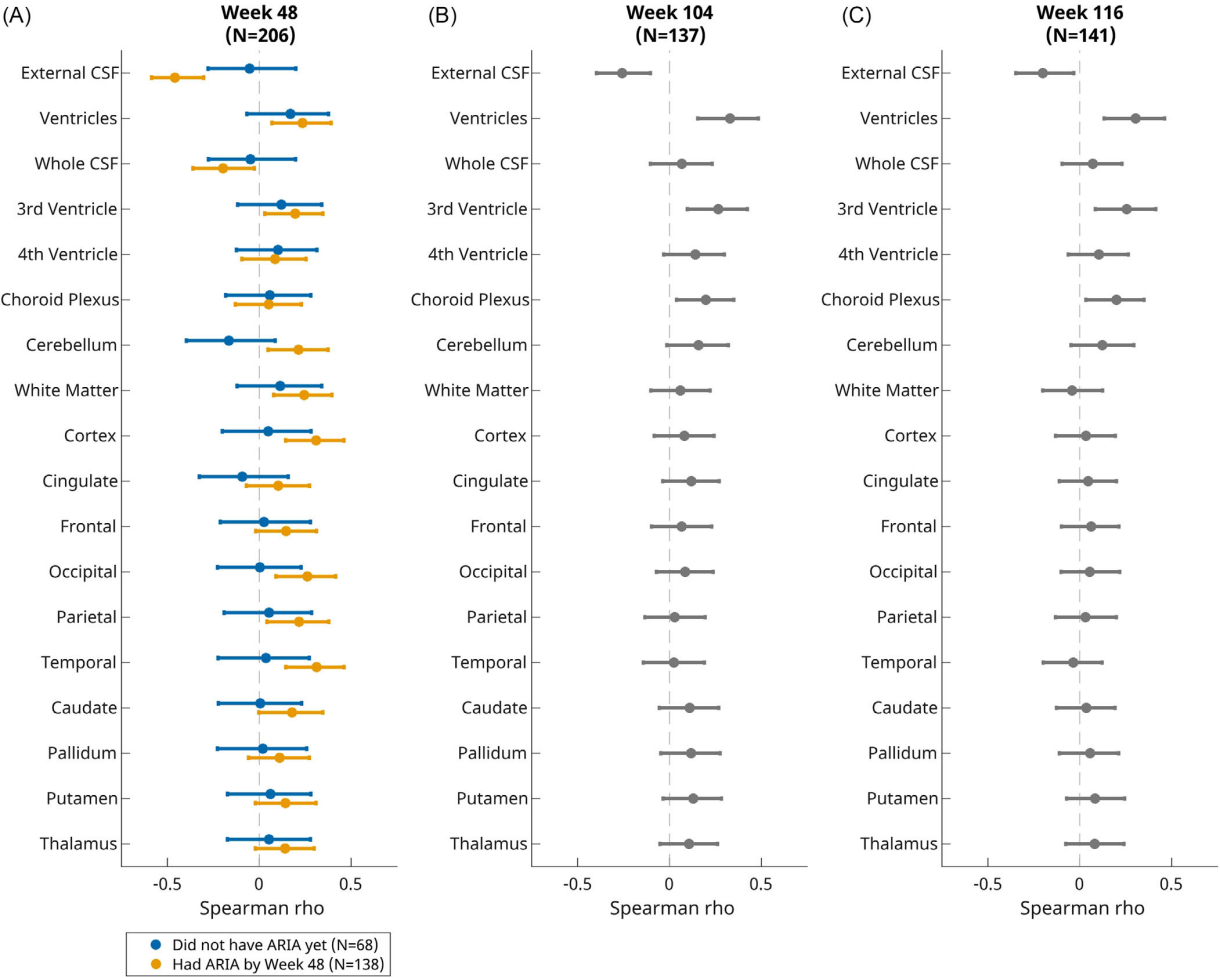
Spearman correlations between regional volume change and the maximum BGTS score in participants with ARIA. The forest plots display the Spearman correlation coefficient (rho) with 95% confidence intervals for various brain regions of interest. The analysis population comprises participants who experienced at least one ARIA event during the study. At the week 48 timepoint, participants were further stratified between those who had not yet experienced an ARIA event by week 48 (blue) and those who had (orange). ARIA, amyloid‐related imaging abnormalities; BGTS, Barkhof Grand Total Score; CSF, cerebrospinal fluid.

At week 48, for the participants who had already experienced an ARIA event, a higher maximum BGTS score (indicating more radiologically severe ARIA) was significantly correlated with *less* volume reduction in the cortex (rho = 0.31; 95% CI, 0.14 to 0.46; *p* < 0.001), white matter (rho = 0.24; 95% CI, 0.08 to 0.40; *p* = 0.004), and cerebellum (rho = 0.21; 95% CI, 0.04 to 0.37; *p* = 0.01). Conversely, for the fluid compartments, a higher maximum BGTS score was associated with greater ventricular enlargement (rho = 0.24; 95% CI, 0.07 to 0.39; *p* = 0.006) but less expansion of the external CSF (rho = −0.46; 95% CI, −0.59 to −0.31; *p* < 0.001). These correlations were not observed in participants who had yet to develop ARIA by week 48 (Figure S8 in supporting information). By the end of the study (i.e., week 116), the associations between maximum BGTS and parenchymal volume reduction became non‐significant, while the correlations with ventricular enlargement and reduced external CSF expansion remained (rho = 0.30; 95% CI, 0.14 to 0.46; *p* < 0.001 and rho = −0.20; 95% CI, −0.36 to −0.04; *p* = 0.02, respectively). However, when evaluated as a composite whole CSF region, the overall volume change did not correlate with the maximum BGTS score at week 116 (rho = 0.07; 95% CI, −0.10 to 0.23; *p* = 0.40; Figure 2).

### 3.4 Association between ventricular and external CSF volume changes

**FIGURE 3:**
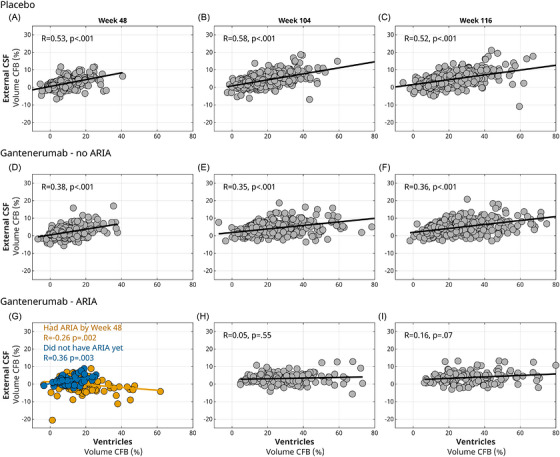
Correlation between ventricular and external CSF volume changes. Scatter plots illustrate the correlation between the percentage change from baseline in ventricular volume (*x* axis) and external CSF volume (*y* axis). The plots are arranged by treatment group (rows: placebo, gantenerumab without ARIA, and gantenerumab with ARIA) and timepoint (columns: week 48, week 104, and week 116). In the gantenerumab with ARIA group at week 48, participants were further stratified between those who had not yet experienced an ARIA event by week 48 (blue) and those who had (orange). ARIA, amyloid‐related imaging abnormalities; CFB, change from baseline; CSF, cerebrospinal fluid.

To further understand the fluid dynamic changes, the correlation between ventricular and external CSF volume changes was analyzed across the three groups (Figure 3). In the placebo group, a clear positive correlation was observed at all timepoints (rho ranging from 0.52–0.58, *p* < 0.001), indicating a parallel expansion of these two compartments consistent with typical disease progression. A similar, though attenuated, positive correlation was present in the gantenerumab‐treated non‐ARIA group (rho ranging from 0.35–0.38, *p* < 0.001). However, this relationship was inverted in the gantenerumab ARIA group (Figure 3G). At week 48, for participants who had an ARIA‐E event by week 48, the correlation became negative (rho = −0.26, 95% CI −0.41 to −0.09, *p* = 0.002); for many of these individuals, ventricular expansion was accompanied by an actual shrinkage of the external CSF space. Instead, participants who did not have an ARIA‐E event at week 48 yet behaved similarly to the non‐ARIA group (rho = 0.36, 95% CI 0.13–0.56, *p* = 0.003). At later time points, the correlation coefficients appeared to revert toward a positive value.

### 3.5 Association between amyloid removal and volume changes

The ARIA and non‐ARIA gantenerumab‐treated groups were merged for subsequent analyses, as no differences in volume change were found between them in any parenchymal region (with the exception of minor differences in the thalamus and cerebellum that exhibited lower volume reduction in the ARIA group compared to the non‐ARIA group).

**FIGURE 4:**
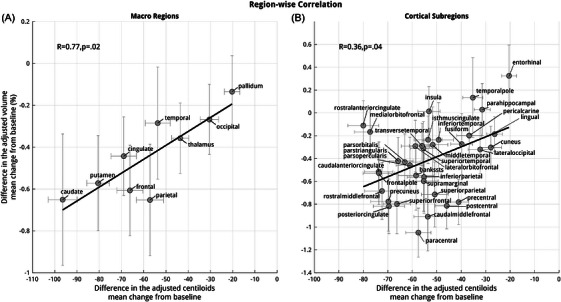
Region‐wise correlation analysis between amyloid removal and volume change. The scatterplots illustrate the correlation between the net treatment effect on amyloid burden (measured in Centiloids) and the net treatment effect on regional brain volume (%) at the end of the study for (A) macro regions and (B) cortical subregions. Each point represents a specific region of interest. The net treatment effect for each axis was calculated using a mixed model for repeated measures (MMRM) as the difference in adjusted mean changes from baseline between the gantenerumab and placebo groups. The solid lines represent the linear regression. R indicates the Spearman correlation coefficient and *p* the corresponding *p* value. Horizontal and vertical bars represent the 95% confidence intervals for amyloid and volume changes, respectively, as obtained from the MMRM.

The region‐wise correlation analysis revealed that the amount of volume reduction relative to placebo strongly correlated with the regional amount of amyloid removed (rho = 0.77, 95% CI 0.19 to 0.95, *p* = 0.02), indicating that brain regions with greater amyloid clearance exhibited greater volume reduction (Figure 4A).

This positive relationship between net treatment effect on brain volume change and amyloid removal was also found when examining a finer level of parcellation restricted to cortical subregions (Figure 4B), at week 48 in both the gantenerumab ARIA and no ARIA groups, as well as in the gantenerumab no ARIA group at week 116 (Figure S9 in supporting information), and remained positive in a sensitivity analysis restricted to participants imaged with [^18^F]florbetaben using regional SUVRs instead of regional Centiloids (Figure S10 in supporting information).

### 3.6 Association between volume changes and clinical outcomes

**FIGURE 5:**
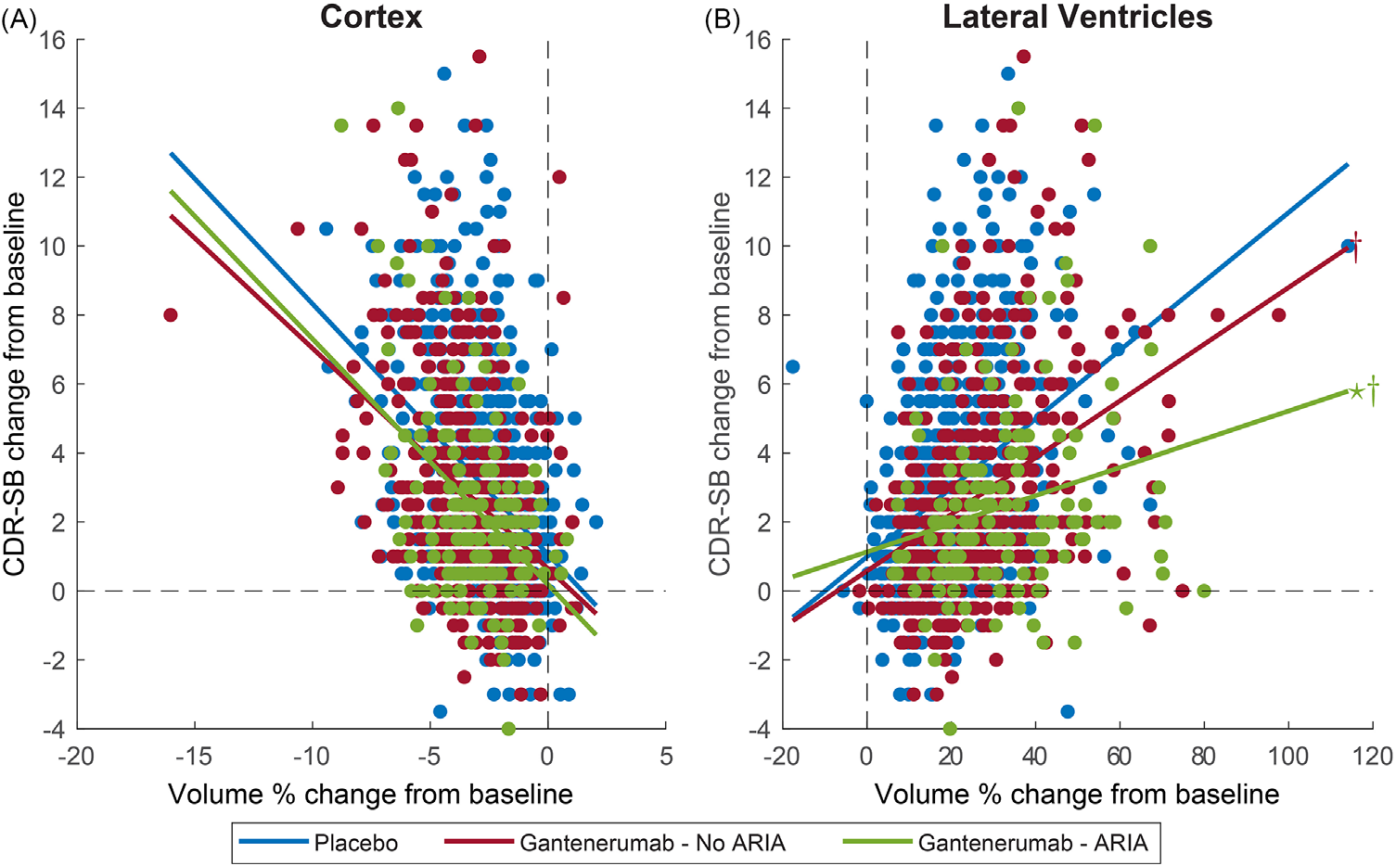
Association between volume changes and clinical outcomes. This figure shows the relationship between cortical and ventricular volume change against Clinical Dementia Rating Sum of Boxes (CDR‐SB) change from baseline. The lines represent the linear model fit in the placebo group (blue), the gantenerumab group without ARIA (red), and the gantenerumab group with ARIA (green). A star (★) denotes a statistically significant difference (*p* < 0.05) for a gantenerumab group compared to the placebo group, while a dagger (†) indicates a significant difference (*p* < 0.05) between the gantenerumab with ARIA and without ARIA groups. ARIA, amyloid‐related imaging abnormalities.

Cortical volume change from baseline was associated with CDR‐SB change from baseline in the placebo group (β = −0.73; 95% CI −0.85 to −0.60; *p* < 0.001), the gantenerumab‐treated non‐ARIA group (β = −0.64; 95% CI −0.77 to −0.51; *p* < 0.001), and the gantenerumab‐treated ARIA group (β = −0.71; 95% CI −0.98 to −0.45; *p* < 0.001), with no statistical differences among the three groups (*F*[2,1219] = 0.47; *p* = 0.62; Figure 5A).

Ventricular volume change from baseline was also associated with CDR‐SB change from baseline in the placebo group (β = 0.10; 95% CI 0.10 to 0.08; *p* < 0.001), the gantenerumab‐treated non‐ARIA group (β = 0.08; 95% CI 0.06 to 0.10; *p* < 0.001), and the gantenerumab‐treated ARIA group (β = 0.04; 95% CI 0.01 to 0.07; *p* = 0.008), with the ARIA group showing a significantly flatter relationship between ventricular expansion and CDR‐SB progression compared to the non‐ARIA group (difference in slope = 0.04; 95% CI 0.01 to 0.08; *p* = 0.02) and placebo (difference in slope = 0.06; 95% CI 0.02 to 0.09; *p* = 0.001; Figure 5B). These findings were consistent for all secondary clinical endpoints (Figure S11 in supporting information).

## 4 DISCUSSION

The volumetric MRI changes observed with gantenerumab appear to be the net result of three distinct processes. The largest driver of volume change remains the underlying disease progression. Superimposed on this are two treatment‐related effects: fluid shifts between ventricular and external CSF spaces driven by ARIA‐E, and small parenchymal volume reductions linked directly to amyloid plaque removal.

It is important to note that the majority of parenchymal volume reduction was driven by disease progression, with the additional treatment‐related effect being comparatively small (i.e., adjusted mean difference vs. placebo of ≈ 0.5% over 116 weeks), consistent with what has been observed in other amyloid‐targeting antibody trials.^1^, ^2^, ^3^, ^5^

### 4.1 Association between ARIA‐E and volume changes

These small, additional parenchymal changes are not related to the occurrence of ARIA‐E, as by the end of the study no brain region showed greater volume reduction in the ARIA group compared to the non‐ARIA group.

While ARIA‐E was not associated with long‐term parenchymal changes, it did have a clear impact on CSF spaces. Consistent with reports for similar molecules,^9^, ^22^ ARIA‐E was associated with ventricular volume expansion. A novel finding of this analysis, however, was that participants with ARIA‐E also showed less expansion of the external CSF space compared to those without ARIA‐E or on placebo.

These two effects were directly linked. The volume changes in the ventricular and external CSF compartments were correlated with the radiological severity of ARIA‐E and to each other, such that participants with more severe ARIA‐E exhibited greater ventricular enlargement alongside a corresponding, greater reduction of the external CSF space. This suggests a redistribution of fluid between the compartments, a hypothesis supported by the finding that the whole (total) CSF expansion did not differ between the ARIA and non‐ARIA groups, indicating that the reduced expansion of the external CSF offsets the additional expansion of the ventricles in the ARIA group. The precise mechanism behind this fluid shift is unknown. One possible hypothesis could be CSF malabsorption due to glymphatic “clogging.”^7^ This obstruction may be caused by the mobilization of soluble Aβ fragments from plaques—a process hypothesized to induce ARIA‐E—^20^, ^23^ and potentially exacerbated by the protein‐rich edema that ARIA‐E itself produces.^24^ It is also possible that the fluid shift is caused by increased CSF production, as the inflammatory nature of ARIA‐E may trigger choroid plexus hypersecretion, as seen in animal models of posthemorrhagic hydrocephalus.^24^ An increased choroid plexus volume was indeed observed in the active arm, especially in the ARIA group, compared to placebo (Figure S3L).

Notably, while ARIA‐E is typically a focal phenomenon, with transient volume changes peaking at the time of the event in regions such as the occipital lobe (Figure S3), the associated fluid shifts in the CSF compartments are diffuse and persist until the end of the study. These diffuse fluid shifts, which include ventricular enlargement and external CSF shrinkage, are radiological findings reminiscent of conditions like communicating or normal pressure hydrocephalus. However, unlike these pathological states which frequently result in overt clinical consequences such as gait impairment and urinary incontinence,^25^ the fluid shifts observed in the context of ARIA‐E appear to remain subclinical and physiologically compensated.

A subtle fluid shift appears to occur even without overt ARIA‐E, as gantenerumab‐treated participants in this group still exhibited greater ventricular expansion than placebo. The positive correlation between ventricular and external CSF volume changes was weakened compared to placebo, suggesting a low‐magnitude effect, strong enough to enlarge the ventricles but too subtle to fully invert the fluid compartment relationship as observed in the ARIA group.

### 4.2 Association between amyloid removal and volume changes

While the effect of ARIA‐E was primarily on CSF compartment volumes, amyloid removal was directly associated with the additional volume change observed in parenchymal regions. This relationship was established by calculating the net, placebo‐corrected drug effect on both volume and amyloid for each parenchymal region, revealing that the additional volume change was greatest in regions with the most significant amyloid removal.

Although these findings support a spatial link between amyloid clearance and volume change, the present data cannot fully elucidate the precise mechanism. The amount (dry weight) of Aβ protein itself has been argued to be insufficient to explain the observed volume differences.^12^ However, amyloid plaques are complex structures that also contain a host of other proteins, and are associated with reactive glia and fluid, all of which occupy volume. One hypothesis is that the removal of this entire composite volume accounts for the macroscopic changes observed in these trials.^8^, ^10^

An alternative hypothesis is that this regional association reflects shared underlying biological processes triggered by local drug activity. *Post mortem* data from individuals receiving active anti‐amyloid immunotherapy have shown reduced neuronal density, which has been hypothesized to occur when microglia activated to remove amyloid plaques also phagocytose neighboring damaged neurons.^26^

### 4.3 Volumetric changes in the white matter and cerebellum

Both the white matter and the cerebellum are expected to have low amyloid burden in an early AD population such as the one enrolled in the GRADUATE trials.^27^ Nonetheless, the white matter showed greater volume reduction in gantenerumab‐treated participants compared to placebo, while the cerebellum showed less volume reduction. The underlying biological mechanisms driving these divergent changes in brain regions with low amyloid pathology are unknown.

Reliably quantifying amyloid removal in these regions is also challenging: the cerebellum serves as the reference region for PET quantification, making its longitudinal change equal to zero, while all current amyloid PET tracers exhibit high, non‐specific off‐target binding in the white matter, making it unclear what biological substrate the tracers quantify in this compartment.^28^ Despite these quantification limitations, the observed volumetric trajectories of these regions currently challenge the hypothesis that macroscopic volume reduction is exclusively linked to the removal of amyloid plaques, suggesting that additional, more complex mechanisms are involved.

### 4.4 Relationship between volume change and clinical outcomes

The gantenerumab ARIA group exhibited a significantly flatter relationship between ventricular expansion and CDR‐SB worsening compared to placebo and the no‐ARIA group, indicating that for a given magnitude of ventricular expansion, these participants experienced less clinical decline. This finding is in line with a previous report from the GRADUATE studies indicating no evidence that ARIA‐E impacted longitudinal cognitive or functional outcomes at the group level.^17^ It is also consistent with broader evidence from amyloid‐targeting immunotherapy trials, in which placebo‐adjusted ventricular volume changes show no correlation with cognitive outcomes,^8^, ^10^ suggesting these fluid shifts are not associated with long‐term functional detriment.

Conversely, no group differences were found in the association between cortical volume reduction and clinical decline. The lack of statistical significance likely reflects the small magnitude of treatment‐associated cortical volume change (≈ 0.5% vs. placebo), which may be insufficient to drive a detectable divergence in clinical correlation.

Importantly, the significant correlation observed between cortical volume change and clinical score change in the treated groups must be interpreted with caution as these simple within‐group correlations are heavily confounded by underlying disease progression, which remains the primary driver of both volume change and cognitive decline across all arms. To properly evaluate if treatment‐induced volume change relates to cognitive worsening, one must correlate the net treatment effect on volume with the net treatment effect on cognition. Calculating this at the individual level is not possible due to the counterfactual nature of clinical trials: a participant is either on the drug or on placebo, so an individual “net effect” cannot be calculated.

The only valid alternative is to evaluate trial‐level data, at which net placebo‐adjusted effects can be calculated per study. These analyses have shown that greater treatment‐induced parenchymal volume reduction is associated with better clinical outcomes,^8^, ^10^ suggesting these changes represent a downstream consequence of amyloid removal rather than neurodegeneration.

### 4.5 Limitations

Some limitations of this study should also be considered. Although gantenerumab treatment resulted in a substantial reduction in amyloid load, most participants remained amyloid positive at the end of the study. This precludes any conclusions about the long‐term volumetric effects after complete amyloid clearance. The analyses involving PET imaging were also constrained by the limited sample size for amyloid PET. Furthermore, the analyses presented here are post hoc and exploratory, and were conducted without formal control for type I errors. Moreover, because the subgroup comparisons were not formally powered, the potential for type II errors in the ARIA versus non‐ARIA analyses cannot be excluded.

### 4.6 Conclusion

In this study, ARIA‐E did not drive long‐term parenchymal volume reduction; its primary impact was a fluid shift within CSF compartments. Conversely, the removal of amyloid was linked to parenchymal volume reduction, with changes corresponding to the magnitude of local amyloid clearance. Furthermore, treated participants showed greater volume reduction in the white matter and less reduction in the cerebellum compared to placebo. Because both regions typically have low amyloid pathology, these findings suggest that additional mechanisms other than local amyloid clearance are involved. Ultimately, these treatment‐associated neuroanatomical changes were not associated with worse clinical/cognitive outcomes.

## CONFLICT OF INTEREST STATEMENT

Matteo Tonietto and Gregory Klein are full‐time employees and own stock in F. Hoffmann‐La Roche Ltd. Erica Silvestri is an external consultant of F. Hoffmann‐La Roche Ltd. Christopher Belder has served on an advisory board for Lilly with honoraria paid to his institution. Frederik Barkhof has served on steering committees or data safety monitoring boards for Biogen, Merck, Eisai, and Prothena. He has served on advisory boards for Combinostics, Scottish Brain Sciences, and Alzheimer Europe. He has provided consultancy services for Roche, Celltrion, Rewind Therapeutics, Merck, and Bracco. He has research agreements with ADDI, Merck, Biogen, GE Healthcare, and Roche. He is co‐founder and shareholder of Queen Square Analytics LTD. Nick Fox has provided consultancy services or served on advisory boards for Abbvie, Biogen, Eisai, Eli Lilly, Ionis, and Roche. Janice Smith is a full‐time employee of Roche Products Ltd. and owns stock in F. Hoffmann‐La Roche Ltd. Author disclosures are available in the Supporting Information.

## CONSENT STATEMENT

The study was conducted in accordance with the Declaration of Helsinki, approved by the relevant institutional ethics committees, and all participants provided written informed consent.

## Supporting information



Supporting Information

Supporting Information
