# Supplementary material for: Volume change with gantenerumab: Impact of amyloid‐related imaging abnormalities and amyloid removal
Source: Alzheimers Dement. 2026 Aug 14;22(8):e71749. doi: 10.1002/alz.71749 (PMC13474159; doi:10.1002/alz.71749)
Supplement: Supplementary file 2 [file ALZ-22-e71749-s002.docx]

**Supplemental Online Content**

**eMethods.** Image Acquisition and Processing

**eTable 1.** List of Regions of Interests

**eTable 2.** ARIA-E Status at the Volumetric MRI Visits in the Gantenerumab-treated ARIA Group

**eFigure 1:** CONSORT Flow Diagram

**eFigure 2.** Population Distribution of Peak ARIA-E Radiological Severity

**eFigure 3.** Effect of Gantenerumab on Longitudinal Brain Volume Changes

**eFigure 4.** Effect of Gantenerumab on Longitudinal Brain Volume Changes in APOE ε4 Non-Carriers (0 Allele)

**eFigure 5.** Effect of Gantenerumab on Longitudinal Brain Volume Changes in APOE ε4 Carriers (1 Allele)

**eFigure 6.** Effect of Gantenerumab on Longitudinal Brain Volume Changes in APOE ε4 Carriers (2 Allele)

**eFigure 7.** Effect of Gantenerumab on Longitudinal Brain Absolute Volume Changes

**eFigure 8.** Association between CSF Compartment Volume Changes and ARIA Radiological Severity

**eFigure 9.** Regional Correlation Between Net Amyloid Plaque Reduction and Brain Volume Changes in Gantenerumab-Treated Participants with and Without ARIA

**eFigure 10.** Regional Correlation Between Net Amyloid Plaque Reduction measured with [¹⁸F]Florbetaben SUVR and Brain Volume Changes in Gantenerumab-Treated Participants

**eFigure 11.** Association between Volume Changes and Clinical Outcomes

**eReferences**

# eMethods: Image Acquisition and Processing

## MRI acquisition

MRI scanners were configured following the Alzheimer’s Disease Neuroimaging Initiative (ADNI) 3 recommended sequences [1] allowing only 1.5 and 3T scanners that had been qualified and harmonized by the imaging core laboratory. 822 of the 1548 participants (53.1%) included in the analysis were imaged on a 3T MRI scanner.

Longitudinal volumetric MRI analyses were restricted to scans acquired at Weeks 48, 104, and 116, as these were the prespecified milestones dedicated to volumetric assessment in the GRADUATE trial protocols. Because of this pre-specification, high-resolution 3D T1-weighted images at these visits underwent a dedicated visual quality control process by the imaging core laboratory to ensure suitability for volumetric analysis, and scans with artifacts were repeated when possible.

## MRI processing

Voxel wise maps of relative volume changes were calculated using the Jacobian integration method as implemented in Statistical Parametric Mapping 12 software [2] (SPM12, version 7771). For each participant, all available visits were used to create a within-subject template using non-linear diffeomorphic registrations. This template captured subject-specific anatomy, ensuring accurate alignment across time points, and it was unbiased with respect to the chronological scan order. Deformation fields were subsequently computed by registering each time point to the within-subject template. The Jacobian determinants of these fields represent the local relative volume change.

To extract regional volume changes from these maps, various regions of interest (ROIs) were defined (see eTable 1 for a complete list). Most of the ROIs were generated by applying the FreeSurfer longitudinal stream (version 6.0) to all available T1-weighted images for each participant, to obtain robust and consistent brain segmentations based on the Desikan-Killiany atlas [3].

Additionally, some regions were further defined using more accurate segmentation methods. These include:

- Lateral ventricles: Lateral ventricular masks were obtained by applying the automatic segmentation toolbox introduced by Fonov and colleagues [4] to the screening T1-weighted image.
- Choroid plexus: Choroid plexus masks were obtained by applying the automatic segmentation toolbox introduced by Yazdan-Panah and colleagues [5] to the screening T1-weighted image.
- External CSF: The external CSF space was obtained as the intersection between the cerebrospinal fluid mask obtained from SPM12 and a 3 mm distance map from the FreeSurfer-derived cortical surface (see eTable 1). Notably, this definition does not encompass the entire CSF space up to the inner skull table. However, this restricted region approximates to that space and optimizes the reliability of the longitudinal Jacobian analysis.
- Whole CSF: The Whole CSF mask was defined as the geometric union of the Lateral Ventricles, Third Ventricle, Fourth Ventricle, the External CSF mask, and the FreeSurfer *aseg* "CSF" region (label 24), the latter encompassing central cerebrospinal fluid volumes not included in standard ventricular segmentations

Finally, to calculate the regional volume trajectories, all the ROI masks defined above were non-linearly registered to the individual within-subject template space by applying the deformation field estimated within the SPM routine.

## Amyloid PET acquisition

Longitudinal amyloid PET scans were acquired in a subset of participants whose amyloid status at screening had been determined by amyloid PET and who provided consent to participate in the longitudinal amyloid PET sub-study. At each visit, participants underwent a total of 20 minutes of serial PET imaging (4 frames of 5 minutes), starting 90 minutes (±1 minute) after an intravenous injection of either 300 MBq (±20%) of [^18^F]florbetaben or 185 MBq (±10%) of [^18^F]flutemetamol. The choice of tracer depended on the availability at each country and site. All PET scanners were calibrated using a Hoffman phantom, and analyses were performed on images with matched spatial resolution, using the methodology introduced by Joshi and colleagues [6].

## Amyloid PET image processing

PET images were preprocessed with PMOD software (PMOD Technologies, Zurich, Switzerland). The workflow included correction for motion artifacts, temporal averaging, and smoothing to achieve a uniform resolution of 7.0 mm in-plane and 8.0 mm axially.

To ensure optimal alignment between longitudinal amyloid PET images, we followed the steps detailed by Schwarz et al., in their “Mayo SUVR pipeline” [7]. In the first step, a longitudinal amyloid PET template was generated using the *buildtemplate* routine from Advanced Normalization Tools (ANTs) [8], and individual visits were linearly registered to this template. Contrary to the MRI within-subject template where non-linear diffeomorphic transformations were employed, this PET within-subject template was created using rigid transformations only. In the second step, the PET within-subject template was aligned to the MRI within-subject template. The two transformations were then concatenated and applied to the harmonized PET images from each visit in a single interpolation step.

Standardized uptake value ratios (SUVRs) were calculated for each region of interest using the whole cerebellum as the reference region (cerebellum ROI in eTable 1). The SUVRs were then converted to centiloids (CL) using tracer-specific global equations derived internally according to the GAAIN calibration guidelines and dataset [9]. Specifically:

For [^18^F]florbetaben:

CL = 167.2 × SUVR_FBB_ − 163.9

For [^18^F]flutemetamol:

CL = 134.7 × SUVR_FMM_ − 129.9

Here, SUVR_FBB_ and SUVR_FMM_ refer respectively to the SUVRs obtained from PET acquisitions using [^18^F]florbetaben and [^18^F]flutemetamol.

While these equations are designed to obtain a global Centiloid value from a global SUVR, their use to calculate regional Centiloid changes from baseline has been recently validated by our group [10]. In brief, we demonstrated that while a regional intercept is required for proper harmonization between amyloid tracers, the global slope of the conversion equation is maintained across regions. Because the present analysis evaluated longitudinal changes from baseline in Centiloids, the regional intercepts mathematically cancel out, making the use of the global slope appropriate to harmonise regional amyloid changes from baseline.

# eTable1. List of Regions of Interests

| **Region** | **Atlas Labels** | **Notes** |
| --- | --- | --- |
| Cortex | All the cortical regions included in the left and right hemisphere *aparc* segmentations. |  |
| Cingulate | ctx-lh-caudalanteriorcingulate, ctx-lh-isthmuscingulate, ctx-lh-posteriorcingulate, ctx-lh-rostralanteriorcingulate, ctx-rh-caudalanteriorcingulate, ctx-rh-isthmuscingulate, ctx-rh-posteriorcingulate, ctx-rh-rostralanteriorcingulate | Defined as in [11] |
| Frontal | ctx-lh-caudalmiddlefrontal, ctx-lh-lateralorbitofrontal, ctx-lh-medialorbitofrontal, ctx-lh-parsopercularis, ctx-lh-parsorbitalis, ctx-lh-parstriangularis, ctx-lh-rostralmiddlefrontal, ctx-lh-superiorfrontal, ctx-lh-frontalpole, ctx-rh-caudalmiddlefrontal, ctx-rh-lateralorbitofrontal, ctx-rh-medialorbitofrontal, ctx-rh-parsopercularis, ctx-rh-parsorbitalis, ctx-rh-parstriangularis, ctx-rh-rostralmiddlefrontal, ctx-rh-superiorfrontal, ctx-rh-frontalpole | Defined as *Frontal regions* in [12] |
| Occipital | ctx-lh-lateraloccipital, ctx-lh-lingual, ctx-lh-cuneus, ctx-lh-pericalcarine, ctx-rh-lateraloccipital, ctx-rh-lingual, ctx-rh-cuneus, ctx-rh-pericalcarine | Defined as in [12] |
| Parietal | ctx-lh-inferiorparietal, ctx-lh-precuneus, ctx-lh-superiorparietal, ctx-lh-supramarginal, ctx-rh-inferiorparietal, ctx-rh-precuneus, ctx-rh-superiorparietal, ctx-rh-supramarginal | Defined as *Lateral parietal regions* in [12] |
| Temporal | ctx-lh-middletemporal, ctx-lh-superiortemporal, ctx-rh-middletemporal, ctx-rh-superiortemporal | Defined as *Lateral temporal regions* in [12] |
| Caudate | Left-Caudate, Right-Caudate | To minimize partial volume effects associated with cerebrospinal fluid, the masks of these four nuclei were refined using a CSF mask that: 1) included the lateral ventricles (as described below), the third and fourth ventricles, as well as the CSF region (label 24 in the *aseg* segmentation) and 2) was dilated using a Gaussian kernel with a sigma value of 2 mm. |
| Pallidum | Left-Pallidum, Right-Pallidum |  |
| Putamen | Left-Putamen, Right-Putamen |  |
| Thalamus | Left-Thalamus-Proper, Right-Thalamus-Proper |  |
| White Matter | Left-Cerebral-White-Matter, Right-Cerebral-White-Matter | The mask was eroded using a spherical kernel with a radius of 3 mm. |
| Cerebellum | Left-Cerebellum-Cortex, Right-Cerebellum-Cortex, Left-Cerebellum-White-Matter, Right-Cerebellum-White-Matter |  |
| Third Ventricle | 3rd-Ventricle |  |
| Fourth Ventricle | 4th-Ventricle |  |
| Lateral Ventricles |  | Obtained applying the lateral ventricle automatic segmentation toolbox introduced by Fonov and colleagues [4] to the screening T1-weighted image. |
| External CSF |  | The external CSF mask, representing the cerebrospinal fluid surrounding the brain up to a distance of 3 mm from the cortical surface, was created using Freesurfer’s *aparc* segmentation to generate a distance map from the cortex into the CSF. This map was thresholded to include distances up to 3 mm and then binarized. To ensure the exclusion of irrelevant regions such as bones or soft tissues, voxels were removed if they were not identified within the SPM-based CSF segmentation (c3, thresholded at 0.6) or were located within 5 mm of the brainstem or cerebellum. |
| Whole CSF |  | The Whole CSF mask represents the comprehensive cerebrospinal fluid volume, calculated as the geometric union of the ventricular system (Lateral Ventricles, Third *Ventricle*, and Fourth Ventricle), the "CSF" region (label 24 in the *aseg* segmentation), and the External CSF mask |
| Choroid Plexus |  | Obtained applying the automatic segmentation toolbox introduced by Yazdan-Panah and colleagues [5] to the screening T1-weighted image. |

# eTable2: ARIA-E Status at the Volumetric MRI Visits in the Gantenerumab-treated ARIA Group

|  | **Week 48** | **Week 104** | **Week 116** |
| --- | --- | --- | --- |
| **No ARIA** (BGTS=0 at current and all previous visits)  **N (%)** | 68 (33.0) | 4 (2.9) | 0 (0) |
| **Active ARIA** (BGTS>0 at current visit)  **N (%)** | 94 (45.6) | 24 (17.5) | 28 (19.9) |
| **Resolved ARIA** (BGTS=0 at current visit, and BGTS>0 at any previous visits)  **N (%)** | 44 (21.4) | 109 (79.6) | 113 (80.1) |
| **Total**  **N** | 206 | 137 | 141 |

**Abbreviations**: ARIA: Amyloid-Related Imaging Abnormalities; BGTS: Barkhof Grand Total Score

# eFigure 1: CONSORT Flow Diagram

Assessed for eligibility

(n= 10907)

Excluded (n= 8874)

Not meeting inclusion criteria (n= 8874)

Randomly assigned to receive gantenerumab (n= 1032)

Received at least one dose (n= 1032)

Randomised (n= 2033)

Analysed (n= 786)

Excluded from analysis (n= 122)

Missing baseline T1-w MRI (n= 1)

Missing post-baseline T1-w MRI (n= 7)

Scanner Change (n= 87)

Analyses pipeline failure (n= 27)

With longitudinal MRI study (n= 908)

Discontinued before post-baseline MRI (n= 124) due to:

Adverse event (n= 18)

Death (n= 4)

Perceived lack of efficacy (n= 1)

Lost to follow-up (n= 0)

Medical decision (n= 4)

Protocol deviation (n=3)

Study terminated by the sponsor (n= 21)

Withdrawal by the participant (n= 62)

Other (n= 11)

With longitudinal MRI study (n= 882)

Discontinued before post-baseline MRI (n= 113) due to:

Adverse event (n= 6)

Death (n= 8)

Perceived lack of efficacy (n= 0)

Lost to follow-up (n=1)

Medical decision (n=2)

Protocol deviation (n=4)

Study terminated by the sponsor (n= 19)

Withdrawal by the participant (n= 57)

Other (n= 16)

Randomly assigned to receive placebo (n= 1001)

Received at least one dose (n= 995)

Did not receive allocated intervention (n= 6)

Analysed (n= 762)

Excluded from analysis (n= 120)

Missing baseline T1-w MRI (n= 6)

Missing post-baseline T1-w MRI (n= 4)

Scanner Change (n= 81)

Analyses pipeline failure (n= 29)

Secondary Data Analysis

Allocation

Follow-Up

Enrolment

**eFigure 1:** Flow diagram of the progress through the phases of participant enrollment, randomization, follow-up, and data analysis for the pooled analysis of Graduate I and II

# eFigure 2. Population Distribution of Peak ARIA-E Radiological Severity

**eFigure 2: Population Distribution of Peak ARIA-E Radiological Severity.** The histogram illustrates the relative frequency of maximum Barkhof Grand Total Scale (maximum BGTS) scores observed in participants who developed Amyloid-Related Imaging Abnormalities with Edema/Effusion (ARIA-E) during gantenerumab treatment. The data is categorized into three clinically relevant severity levels: Mild (scores <=3; light yellow), Moderate (scores 4–14; teal), and Severe (scores >=15; muted purple). Vertical dashed lines demarcate the categorical transitions boundaries, highlighting the prevalence of mild-to-moderate radiological findings within the treated cohort.

# eFigure 3: Effect of Gantenerumab on Longitudinal Brain Volume Changes

**eFigure 3:** This figure shows the adjusted mean percentage change from baseline with 95% confidence intervals for various brain region volumes over 116 weeks, obtained from the MMRM analysis. The lines represent the Placebo group (blue), the Gantenerumab group without ARIA (red), and the Gantenerumab group with ARIA (green). A star (★) denotes a statistically significant difference (p<.05) for a Gantenerumab group compared to the Placebo group, while a dagger (†) indicates a significant difference (p<.05) between the Gantenerumab with ARIA and without ARIA groups. The embedded table provides the baseline mean and standard deviation normalized volume, which is expressed as a percentage of the estimated total intracranial volume.

# eFigure 4. Effect of Gantenerumab on Longitudinal Brain Volume Changes in APOE ε4 Non-Carriers (0 Alleles)

**eFigure 4: Adjusted Mean Percentage Change in Brain Region Volumes for APOE ε4 Non-Carriers (0 Alleles).** This figure illustrates the adjusted mean percentage change from baseline, including 95% confidence intervals, for specific brain regions over a 116-week period. Data are derived from a mixed model for repeated measures (MMRM) analysis at the group level, specifically stratified for participants possessing zero APOE ε4 alleles. The lines represent the Placebo group (blue), the Gantenerumab group without ARIA (red), and the Gantenerumab group with ARIA (green). A star (★) denotes a statistically significant difference (p<.05) for a Gantenerumab group compared to the Placebo group, while a dagger (†) indicates a significant difference (p<.05) between the Gantenerumab with ARIA and without ARIA groups. The embedded table provides the baseline mean and standard deviation normalized volume, which is expressed as a percentage of the estimated total intracranial volume.

# eFigure 5. Effect of Gantenerumab on Longitudinal Brain Volume Changes in APOE ε4 Carriers (1 Allele)

**eFigure 5: Adjusted Mean Percentage Change in Brain Region Volumes for APOE ε4 Carriers (1 Allele).** This figure illustrates the adjusted mean percentage change from baseline, including 95% confidence intervals, for specific brain regions over a 116-week period. Data are derived from a mixed model for repeated measures (MMRM) analysis at the group level, specifically stratified for participants possessing a single APOE ε4 allele. The lines represent the Placebo group (blue), the Gantenerumab group without ARIA (red), and the Gantenerumab group with ARIA (green). A star (★) denotes a statistically significant difference (p<.05) for a Gantenerumab group compared to the Placebo group, while a dagger (†) indicates a significant difference (p<.05) between the Gantenerumab with ARIA and without ARIA groups. The embedded table provides the baseline mean and standard deviation normalized volume, which is expressed as a percentage of the estimated total intracranial volume.

# eFigure 6. Effect of Gantenerumab on Longitudinal Brain Volume Changes in APOE ε4 Carriers (2 Alleles)

**eFigure 6: Adjusted Mean Percentage Change in Brain Region Volumes for APOE ε4 Carriers (2 Alleles).** This figure illustrates the adjusted mean percentage change from baseline, including 95% confidence intervals, for specific brain regions over a 116-week period. Data are derived from a mixed model for repeated measures (MMRM) analysis at the group level, specifically stratified for participants possessing two APOE ε4 alleles. The lines represent the Placebo group (blue), the Gantenerumab group without ARIA (red), and the Gantenerumab group with ARIA (green). A star (★) denotes a statistically significant difference (p<.05) for a Gantenerumab group compared to the Placebo group, while a dagger (†) indicates a significant difference (p<.05) between the Gantenerumab with ARIA and without ARIA groups. The embedded table provides the baseline mean and standard deviation normalized volume, which is expressed as a percentage of the estimated total intracranial volume.

# eFigure 7. Effect of Gantenerumab on Longitudinal Brain Absolute Volume Changes

**eFigure 7: Adjusted Mean Absolute Change in Brain Region Volumes.** This figure illustrates the adjusted mean absolute change from baseline (in cm^3^), including 95% confidence intervals, for specific brain regions over a 116-week period. Data are derived from a mixed model for repeated measures (MMRM) analysis at the group. The lines represent the Placebo group (blue), the Gantenerumab group without ARIA (red), and the Gantenerumab group with ARIA (green). A star (★) denotes a statistically significant difference (p<.05) for a Gantenerumab group compared to the Placebo group, while a dagger (†) indicates a significant difference (p<.05) between the Gantenerumab with ARIA and without ARIA groups. The embedded table provides the baseline mean and standard deviation volume of each region.

# eFigure 8. Association between CSF Compartment Volume Changes and ARIA Radiological Severity

**eFigure 8: Correlation between external CSF, ventricles, and whole CSF volume changes and the maximum BGTS score in participants with ARIA.** Scatter plots illustrate the correlation between the percentage change from baseline in volume (x-axis) and maximum BGTS score (y-axis). The plots are arranged by regions (rows: external CSF, ventricles, and whole CSF) and timepoint (columns: Week 48, Week 104, and Week 116). At Week 48, participants were further stratified between those who had not yet experienced an ARIA event by week 48 (blue) and those who had (orange).

# eFigure 9. Regional Correlation Between Net Amyloid Plaque Reduction and Brain Volume Changes in Gantenerumab-Treated Participants with and Without ARIA

**eFigure 9:** **Regional Correlation of Treatment Effects on Amyloid Burden and Brain Volume.** The scatterplot illustrates the correlation between the net treatment effect on amyloid burden (measured in Centiloids) and the net treatment effect on regional brain volume (%) at Week 48 (Panels A and C) and Week 116 (Panels B and D). Data are stratified by treated participants who experienced ARIA (top row: Panels A and B) and those who did not (bottom row: Panels C and D). Each point represents a specific region of interest. The net treatment effect for each axis was calculated using a Mixed Model for Repeated Measures (MMRM) as the difference in adjusted mean changes from baseline between the gantenerumab and placebo groups. Solid lines represent the linear regression. R indicates the Spearman correlation coefficient and p the corresponding p-value. Horizontal and vertical bars represent the 95% confidence intervals for amyloid and volume changes, respectively, as obtained from the MMRM.

# eFigure 10. Regional Correlation Between Net Amyloid Plaque Reduction measured with [¹⁸F]florbetaben SUVR and Brain Volume Changes in Gantenerumab-Treated Participants

**eFigure 10:** **Regional Correlation of Treatment Effects on Amyloid Burden and Brain Volume.** The scatterplot illustrates the correlation between the net treatment effect on amyloid burden (measured as SUVR) and the net treatment effect on regional brain volume (%) at the end of the study. Each point represents a specific region of interest. The net treatment effect for each axis was calculated using a Mixed Model for Repeated Measures (MMRM) as the difference in adjusted mean changes from baseline between the gantenerumab and placebo groups. For amyloid PET, the analysis was restricted to participants whose PET scans were acquired using the [^18^F]florbetaben ligand. The solid line represents the linear regression. R indicates the Spearman correlation coefficient and p the corresponding p-value. Horizontal and vertical bars represent the 95% confidence intervals for amyloid and volume changes, respectively, as obtained from the MMRM.

# eFigure 11. Association between Volume Changes and Clinical Outcomes

**eFigure 11: Association between volume changes and clinical outcomes.** This figure shows the relationship between cortical and ventricular volume change against clinical scores change from baseline. The lines represent the linear model fit in the placebo group (blue), the gantenerumab group without ARIA (red), and the gantenerumab group with ARIA (green). A star (★) denotes a statistically significant difference (p<.05) for a gantenerumab group compared to the placebo group, while a dagger (†) indicates a significant difference (p<.05) between the gantenerumab with ARIA and without ARIA groups. ADAS-Cog 13, Alzheimer's Disease Assessment Scale - Cognitive Subscale 13; ADCS-ADL, Alzheimer's Disease Cooperative Study - Activities of Daily Living; FAQ, Functional Activities Questionnaire.

# eReferences

[1]	Weiner MW, Veitch DP, Aisen PS, Beckett LA, Cairns NJ, Green RC, et al. The Alzheimer’s Disease Neuroimaging Initiative 3: Continued innovation for clinical trial improvement. Alzheimers Dement 2017;13:561–71. https://doi.org/10.1016/j.jalz.2016.10.006.

[2]	Ashburner J, Ridgway GR. Symmetric diffeomorphic modeling of longitudinal structural MRI. Front Neurosci 2013;6:1–19. https://doi.org/10.3389/fnins.2012.00197.

[3]	Reuter M, Schmansky NJ, Rosas HD, Fischl B. Within-subject template estimation for unbiased longitudinal image analysis. NeuroImage 2012;61:1402–18. https://doi.org/10.1016/j.neuroimage.2012.02.084.

[4]	Fonov VS, Narayanan S, Arnold DL, Collins DL. Lateral ventricle segmentation based on fusion of expert priors in AD. Proc Intl Soc Mag Reson Med 2010;18:4255.

[5]	Yazdan-Panah A, Schmidt-Mengin M, Ricigliano VAG, Soulier T, Stankoff B, Colliot O. Automatic segmentation of the choroid plexuses: Method and validation in controls and patients with multiple sclerosis. NeuroImage Clin 2023;38. https://doi.org/10.1016/j.nicl.2023.103368.

[6]	Joshi A, Koeppe RA, Fessler JA. Reducing between scanner differences in multi-center PET studies. NeuroImage 2009;46:154–9. https://doi.org/10.1016/j.neuroimage.2009.01.057.

[7]	Schwarz CG, Therneau TM, Weigand SD, Gunter JL, Lowe VJ, Przybelski SA, et al. Selecting software pipelines for change in flortaucipir SUVR: Balancing repeatability and group separation. NeuroImage 2021;238:118259. https://doi.org/10.1016/j.neuroimage.2021.118259.

[8]	Avants BB, Tustison NJ, Song G, Cook PA, Klein A, Gee JC. A reproducible evaluation of ANTs similarity metric performance in brain image registration. NeuroImage 2011;54:2033–44. https://doi.org/10.1016/j.neuroimage.2010.09.025.

[9]	Klunk WE, Koeppe RA, Price JC, Benzinger TL, Devous MD, Jagust WJ, et al. The Centiloid project: Standardizing quantitative amyloid plaque estimation by PET. Alzheimers Dement 2015;11:1-15.e4. https://doi.org/10.1016/j.jalz.2014.07.003.

[10]	Silvestri. Harmonizing amyloid PET tracers at the regional level: evaluation of regional centiloid equations. Hum Amyloid Imaging 2026. Available at: https://hai.worldeventsforum.com/2026-program-book/.

[11]	Klein A, Tourville J. 101 Labeled Brain Images and a Consistent Human Cortical Labeling Protocol. Front Neurosci 2012;6. https://doi.org/10.3389/fnins.2012.00171.

[12]	Landau S, Ward TJ, Murphy A, Jagust W. Flortaucipir ( AV-1451 ) processing methods 2021. Available at: https://adni.bitbucket.io/reference/docs/UCBERKELEYAV45/ADNI_AV45_Methods_JagustLab_01.20.15.pdf
